# Cloning and characterization of short‐chain *N*‐acyl homoserine lactone‐producing *Enterobacter asburiae* strain L1 from lettuce leaves

**DOI:** 10.1002/mbo3.610

**Published:** 2018-07-07

**Authors:** Yin Yin Lau, Kah Yan How, Wai‐Fong Yin, Kok‐Gan Chan

**Affiliations:** ^1^ Division of Genetics and Molecular Biology Institute of Biological Sciences Faculty of Science University of Malaya Kuala Lumpur Malaysia; ^2^ International Genome Centre Jiangsu University Zhenjiang China; ^3^ ISB Faculty of Science University of Malaya Kuala Lumpu Malaysia

**Keywords:** AHL synthase, biofilm, *Enterobacter asburiae*, *N*‐acyl homoserine lactone, protein expression, quorum sensing

## Abstract

In gram‐negative bacteria, bacterial communication or quorum sensing (QS) is achieved using common signaling molecules known as *N*‐acyl homoserine lactones (AHL). We have previously reported the genome of AHL‐producing bacterium, *Enterobacter asburiae* strain L1. In silico analysis of the strain L1 genome revealed the presence of a pair of *luxI/R* genes responsible for AHL‐type QS, designated as *easIR*. In this work, the 639 bp *luxI* homolog, encoding 212 amino acids, have been cloned and overexpressed in *Escherichia coli* BL21 (DE3)pLysS. The purified protein (~25 kDa) shares high similarity to several members of the LuxI family among different *E* *asburiae* strains. Our findings showed that the heterologously expressed EasI protein has activated violacein production by AHL biosensor *Chromobacterium violaceum* CV026 as the wild‐type *E*. *asburiae*. The mass spectrometry analysis showed the production of *N*‐butanoyl homoserine lactone and *N*–hexanoyl homoserine lactone from induced *E*. *coli* harboring the recombinant EasI, suggesting that EasI is a functional AHL synthase. *E*. *asburiae* strain L1 was also shown to possess biofilm‐forming characteristic activity using crystal violet binding assay. This is the first report on cloning and characterization of the *luxI* homolog from *E*. *asburiae*.

## 1 INTRODUCTION

Consumers’ demand for healthy, garden‐fresh, natural, and convenience food have been on the rise. The lifestyle changing of consumers’ eating habit toward convenient, ready‐to‐eat food products at the meantime has accelerated the incidence and outbreaks of food‐borne diseases worldwide (Berger et al., 2010; Heaton & Jones, 2008). Extensive studies have shown that microbiological contamination of food products is largely due to the naturally occurring phenomenon of biofilm formation. This biofilm‐forming characteristic was found to be correlated with cell‐to‐cell communication, known as quorum sensing (QS). This mechanism is achieved via small diffusible chemical signaling molecules, referred as autoinducers (Chen et al., 2002), to mediate group‐coordinated behaviors (Miller & Bassler, 2001).

In QS, a family of autoinducers known as *N*‐acyl homoserine lactones (AHLs) is widely used as the signaling molecules among Proteobacteria (Miller & Bassler, 2001). AHLs are highly conserved signaling molecules, each consisting of a homoserine lactone ring unsubstituted in the β‐ and γ‐positions but *N*‐acylated at the α‐position with a fatty acyl group. The latter moiety comprises 4‐ to 18‐carbon side chain and either an oxo, a hydroxy, or no substitution at the C3 position (Pearson, Van Delden, & Iglewski, 1999). AHLs are products of the enzyme, AHL synthase (LuxI homolog). They are synthesized using *S*‐adenosylmethionine (SAM) and acylated acyl carrier protein (Acyl‐ACP) as the substrates (Schauder & Bassler, 2001; Swift et al., 1997). When the AHLs concentration has reached its threshold level, the AHLs bind to their cognate receptor (LuxR homolog), resulting in a complex that stimulates the expression of numerous downstream target genes and hence the physiological functions of the cells (How et al., 2015; Parsek & Greenberg, 2000). It was found that the biochemical mechanisms of AHLs synthesis and regulation are conserved in many bacterial species even though they show expression of different phenotypes (Dong, Gusti, Zhang, Xu, & Zhang, 2002).

Over the years, *Enterobacter* spp. have been recognized as increasingly important pathogens (Sanders & Sanders, 1997) and they are commonly isolated from food, water, and soil. The genus can be categorized as pathogenic, opportunistic, or saprophytic bacteria. In fact, *Enterobacter* species are the most frequent bacterial isolates recovered from both inpatient and outpatient clinical specimens. According to Centers for Disease Control and Prevention (CDC), USA and National Nosocomial Infections Surveillance (NNIS) system, this group comprises 30% and 34% of pathogens isolated from infection sites, respectively (Mayhall, 2004). Hence, studies on the potential role of QS system in food‐borne or plant‐associated *Enterobacter*, which are not widely studied, would provide important insight to control the expression of virulence factors.

Recently, a novel AHL‐producing *Enterobacter asburiae* strain L1 has been isolated from lettuce leaves and its genome was completely sequenced by PacBio sequencing platform. This isolate was found to secrete *N*‐butanoyl homoserine lactone (C4‐HSL) and *N*–hexanoyl homoserine lactone (C6‐HSL) (Lau, Sulaiman, Chen, Yin, & Chan, 2013; Lau, Yin, & Chan, 2014). Previous studies have shown that *E*. *asburiae* strains have been isolated from water, soil, food, and human sources, that is, blood culture, wounds, and exudates as well as from respiratory sources (Brenner, McWhorter, Kai, Steigerwalt, & Farmer, 1986; Castellanos‐Arévalo, Castellanos‐Arévalo, Camarena‐Pozos, Colli‐Mull, & Maldonado‐Vega, 2015; Khalifa, Alsyeeh, Almalki, & Saleh, 2015; Shin et al., 2007). Although its exact clinical value is yet to be discovered and very little is known about its pathogenicity and virulence, these findings suggested the clinical significance of *E. asburiae*.

Our previous analysis of the complete genome of *E. asburiae* strain L1 led us to the identification of putative homolog of *luxI* gene. In this study, we deciphered the genomic architecture of strain L1 for autoinducer synthase gene, followed by the molecular characterization of this gene, designated as *easI*. Besides, we also performed comparative genome analysis of strain L1 with other closely related *E. asburiae* strains. The availability of the complete genome of strain L1 and characterization of *easI* gene provide a platform for the functional study of QS and its regulatory role in strain L1.

## 2 MATERIALS AND METHODS

### 2.1 Bacterial strains and culturing conditions

Bacterial strains and plasmids used are described in Table S1. All the bacterial strains were grown aerobically on Luria‐Bertani (LB) broth or agar at 37°C. *Escherichia coli* strains DH5α and BL21 (DE3)pLysS were used for cloning and recombinant protein expression purposes, respectively. When necessary, the transformed cells were grown in LB supplemented with antibiotics in the following concentrations: 100 μg/ml ampicillin (Sigma, USA), 30 μg/ml kanamycin, or 34 μg/ml chloramphenicol (Sigma, USA).

### 2.2 DNA manipulations

The genomic DNA of *E. asburiae* strain L1 DNA was extracted using Masterpure^™^ DNA purification kit (Epicenter, Illumina Inc., USA), whereas plasmids were isolated using QIAprep Spin Miniprep Kit (Qiagen, Germany) as recommended by the manufacturers. The autoinducer synthase gene, *easI*, was amplified from the strain L1 genome, using the following primers: Forward primer, *easI*‐F‐NcoI (5′ CCATGGCGATGAATTCTGTTATTGAGT 3′) and reverse primer, *easI*‐R‐BamHI (5′ GGATCCTAAGTGGCGTAAATGCTCC 3′). The NcoI and BamHI restriction sites were underlined in the primer sequences. Two nonspecific bases CG were added to the forward primer to accommodate the frameshift of the recombinant gene sequence. Polymerase chain reaction (PCR) was performed using Q5^®^ High‐Fidelity DNA polymerase (NEB, USA), programmed to the following condition: initial denaturation at 98°C for 30 s, followed by 27 cycles of 98°C for 10 s, annealing at 55.5°C for 30 s, extension at 72°C for 30 s, a final extension at 72°C for 2 min and a hold temperature at 4°C at the end. After PCR, the amplicon was purified using QIAmp^®^ gel extraction kit (Qiagen, Germany), before subjecting to ligate into a pGEM^®^‐T vector (Promega, USA) per manufacturer's instructions. The resultant recombinant plasmid (designated pGEM^®^‐T‐*easI*) was chemically transformed into *E*. *coli* DH5α (Sambrook & Russel, 2001). Upon confirmation of the recombinants with blue‐white colony screening and colony PCR, the *easI* gene was excised from the plasmid by digestion with FastDigest NcoI and BamHI (Thermo Scientific, USA), followed by ligation with linearized overexpression vector, pET28a (Novagen, Germany) to produce pET28a‐*easI*. The DNA sequence of the constructed plasmids was verified by automated Sanger sequencing.

### 2.3 Gene annotation and comparative genome analysis

Gene annotation for the genome of strain L1 and sequence‐based comparative analysis with other closely related strains was performed using SEED‐based automated annotation system provided by the Rapid Annotations using Subsystems Technology (RAST) server version 4.0 (Aziz et al., 2008). The genome of strain L1 was used as the reference and was compared with the genome of strains PDN3 (JUGH00000000.1), GN02073 (LDCE01000001.1), GN02127 (LDCH00000000.1), and 33838 (LAAP00000000.1), which were obtained from NCBI database (http://www.ncbi.nlm.nih.gov).

### 2.4 Bioinformatics analysis

The nucleotide sequence of *easI* was retrieved from RAST server version 4.0 (Aziz et al., 2008) using “Genome Browser” function. By omitting redundant or ambiguous sequences, the amino acid sequence of EasI was then compared with 14 different strains of *E. asburiae* which possess LuxI homologs, selected from the GenBank database. Multiple sequence alignments of the amino acid sequences were performed using Clustal OMEGA tool with default parameter settings. A neighbor‐joining phylogenetic tree of the *easI* gene was constructed from the aligned sequences using MEGA version 6.0 (Tamura, Stecher, Peterson, Filipski, & Kumar, 2013), whereby the bootstrap has been set to 1,000 repeats. Meanwhile, searches for ORF and prediction of nucleotide translational products were performed using the ORF Finder tool, whereas the fundamental properties of the proteins were predicted using ExPASy.

### 2.5 Heterologous expression of EasI protein in *E*. *coli* and His‐Tagged protein purification

*Escherichia coli* BL21 (DE3)pLysS (Novagen, Germany) harboring pET28a‐*easI* was grown to an OD_600_ of 0.4–0.5 before isopropyl‐β‐D‐thiogalactopyranoside (IPTG) was added at a final concentration of 1.0 mmol/L to induce the expression of the *easI* gene. The cells were further incubated for 8 hr at 25°C, agitated at 250 rpm before harvested by centrifugation at 9391 × *g*. *Escherichia coli* harboring pET28a alone was used as the negative control. Then, the cells were lysed by BugBuster^®^ Protein Extraction Reagent (Novagen, Germany) supplemented with protease inhibitor cocktail (Thermo Scientific, Pittsburgh, PA, USA). The concentration of the protein was determined using Quick Start^™^ Bradford Protein Assay (Bio‐Rad, USA). The His‐tagged protein was purified from the cell lysate using Ni‐NTA Fast Start Kit (Qiagen, Germany) according to the default protocols.

### 2.6 Sodium dodecyl sulfate polyacrylamide gel electrophoresis (SDS‐PAGE) analysis

The protein samples were incubated for 3 min at 95°C before subjected to 12% Tris‐Glycine polyacrylamide gel electrophoresis (Thermo Scientific, USA) using 1× Tris‐Glycine‐SDS buffer (Thermo Scientific, USA) at 125 V for 15 min followed by 150 V for 60 min. To visualize the protein bands, the gels were stained with Coomassie brilliant blue R250 (CBB; Bio‐Rad, USA) for 5 min, followed by destaining step with destaining solution (10% v/v glacial acetic acid; 40% v/v methanol; 50% v/v distilled water) for three times, each 10 min long.

### 2.7 AHL detection, extraction, and identification

The induced *E. coli* BL21 (DE3)pLysS harboring pET28a‐*easI* was screened for AHL production using cross‐streaking with *Chromobacterium violaceum* CV026 (McClean et al., 1997). *Pectobacterium carotovorum* GS101 was used as the positive control, whereas *P. carotovorum* PNP22 (Jones et al., 1993) and *E*. *coli* harboring pET28a alone as the negative controls. The AHL was extracted from the bacterial culture supernatants using a previously reported method but with some modifications (Lau et al., 2013). Briefly, the bacteria cells were incubated overnight for 18 hr in 100 ml LB media with 50 mmol/L 3‐[*N*‐morpholino] propanesulfonic acid (MOPS) buffered to pH 5.5 to prevent spontaneous degradation of AHLs (Yates et al., 2002). The culture was grown at 37°C with shaking at 220 rpm. The spent culture supernatant of the bacterial culture was then extracted thrice with an equal volume of acidified (0.1% v/v glacial acetic acid) ethyl acetate (Merck, Germany) after induction with IPTG. Extracted AHL was left to dry before reconstituted in acetonitrile. The AHL samples were then subjected to LC–MS/MS analysis according to a previously reported method (Lau et al., 2013). An Agilent 1290 Infinity LC system (Agilent Technologies, Santa Clara, CA, USA) equipped with an Agilent ZORBAX Rapid Resolution High Definition SB‐C18 Threaded Column (2.1 mm × 50 mm, 1.8 μm particle size) was used. Precursor ion‐scanning analysis were performed in positive ion mode with Q3 set to monitor for *m*/*z* 102 and Q1 set to scan a mass range of *m*/*z* 80 to *m*/*z* 400. Molecular mass of *m*/*z* 102 refers to the presence of lactone ring, thus indicating presence of AHLs. The MS parameters were as followed: probe capillary voltage set at 3 kV, sheath gas at 11 ml/hr, nebulizer pressure of 20 psi and desolvation temperature at 200°C. Acetonitrile and AHL extract from culture supernatant of *E*. *coli* harboring pET28a alone were used as the blank and negative controls, respectively. All experiments were performed in triplicates.

### 2.8 Biofilm formation and quantification

Biofilm formation and quantification of strain L1 was performed as described by O'Toole (2011) with some modifications. In brief, the overnight culture was diluted with LB medium and the OD_600 was_ adjusted to 0.1. Strain L1 cells were incubated statically for 72 hr at 37°C in 6‐well plate. *Pseudomonas aeruginosa* PAO1 and blank LB medium were used as the positive and negative control, respectively. After incubation, unattached planktonic bacteria were discarded, and the biofilms were washed thrice with sterile distilled water and allowed to air‐dry. To stain the biofilm, 1 ml of crystal violet of 1.0% (v/v) was added to each well for 45 min. The stained biofilms were washed thrice with 1 ml of sterile distilled water, prior to addition of 1 ml of 95% (v/v) ethanol to solubilize the crystal violet. Lastly, 200 μl of the resulting solution was placed into a new microtiter plate for absorbance reading at 590 nm. All experiments were performed in triplicate. The statistical significance of each test (*n* = 3) was evaluated using unpaired *t* test and GraphPad Prism software; a *p* value of ≤.01 was considered significant.

## 3 RESULTS

### 3.1 Gene annotation of strain L1 and comparative genome analysis

**Figure 1:**
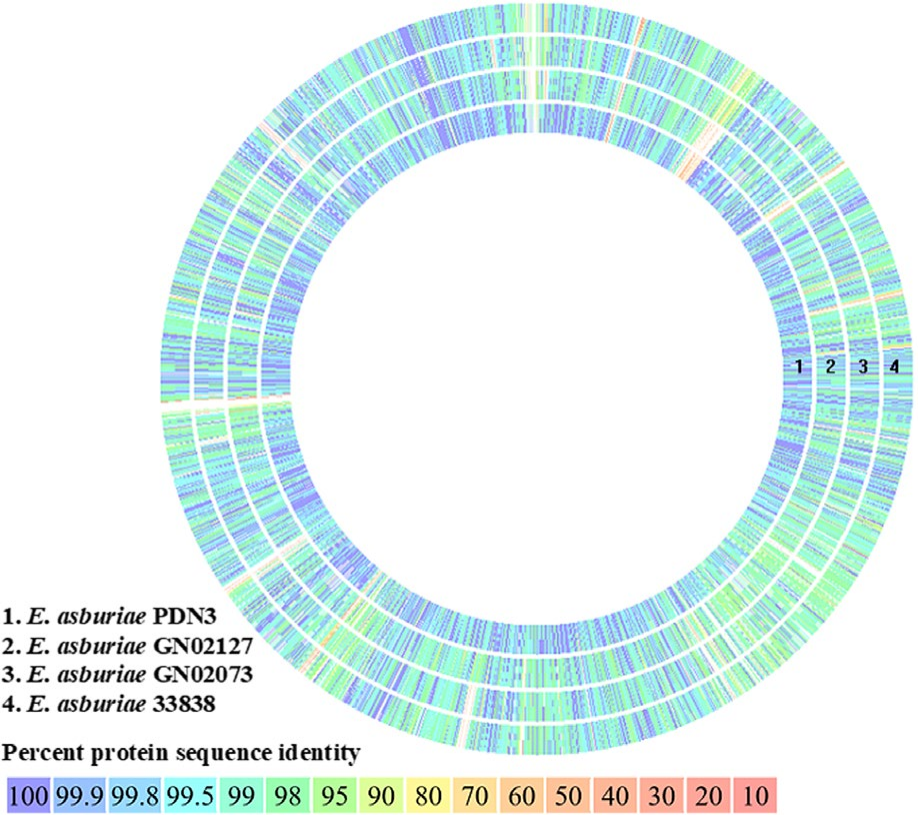
Genome comparison of strain L1 (reference) with four closely related species, PDN3 (JUGH00000000.1), GN02073 (LDCE01000001.1), GN02127 (LDCH00000000.1), and 33838 (LAAP00000000.1) using RAST server. The genome of the reference strain is not displayed in the figure

In previous work by Lau et al. (2014), the whole‐genome sequencing of *E*. *asburiae* strain L1 was performed using PacBio sequencing platform (Pacific Biosciences, Menlo Park, CA, USA). The complete genome sequence of strain L1 has been deposited in DDBJ/EMBL/GenBank under the accession number CP007546. Annotations by RAST revealed that strain L1 and other closely related *E*. *asburiae* strains possess a high abundance of coding DNA sequence (CDS), mainly coding for carbohydrate and amino acids and derivatives (Table S2). Basically, these genes are responsible for the basic physiological functions of the bacterial cells. Sequence‐based comparative analysis of strain L1 with fourteen closely related strains revealed that *E*. *asburiae* strains GN02073, GN02127, 33838 and PDN3 showed high similarities with strain L1, with more than 90% (Figure 1). This strongly indicates that these *E*. *asburiae* strains are likely to have very close genotypic features with each other.

### 3.2 Bioinformatics analysis

**Figure 2:**
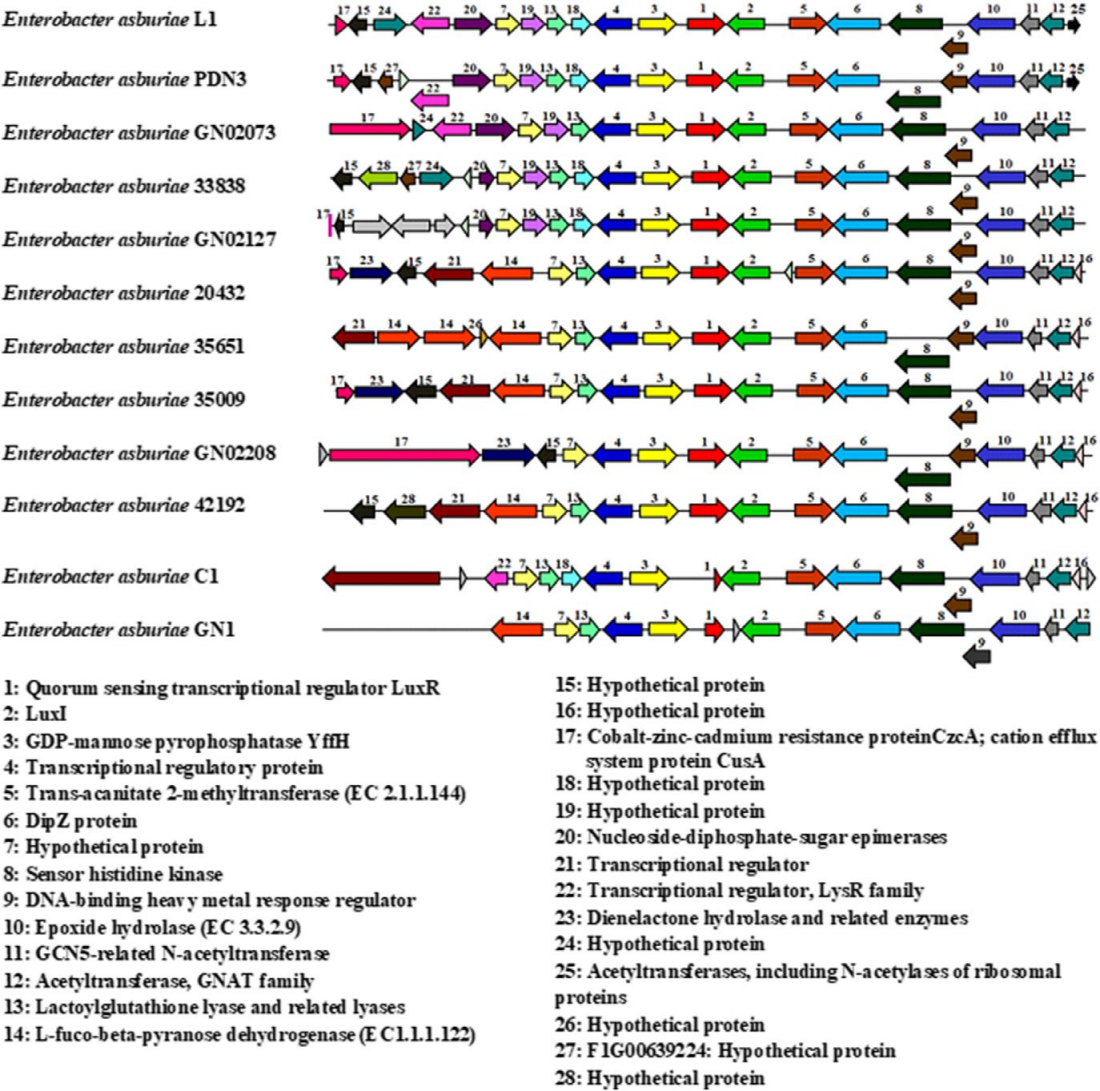
Comparison of *luxI/R* homologs gene clusters in strain L1 and other closely related strains, *Enterobacter asburiae* PDN3, GN02073, 33838, GN02127, 20432, 35651, 35009, GN02208, 42192, C1, and GN1. The relative orientations of the genes are indicated by arrows. The genes which are located outside of the line indicate overlapping genes. Homologous proteins are shown with the same color. Both LuxI autoinducer synthase proteins and transcriptional regulators, LuxRs are found in each strain, except for strains C1 and GN1, which possess truncated *luxR* homologs

Further analysis of strain L1 genome emphasizes on CDS responsible for cell‐to‐cell communication system in *Enterobacter* sp. and we found ORFs coding for putative *luxI* and *luxR* homologs, designated *easI* and *easR* (GenBank accession numbers AHW94257.1 and AHW94256.1, respectively). In Figure 2, a conserved variation is noticeable in the *luxI* gene clusters of strain L1 with other closely related strains. All the *E*. *asburiae* strains possess *luxI* homologs (ORF2) and the convergently transcribed transcriptional regulator *luxR* homologs (ORF1), except for strains C1 and GN1, which possess truncated *luxR* homologs. In the vicinity of the *luxI/R* genes are GCN5‐related N‐acetyltransferase (ORF 11) and acetyltransferase GNAT family (ORF12). Apart from that, a signal‐recognition protein, sensor histidine kinase (ORF8) is found at the downstream of *luxI* homologs.

Based on NCBI database, the 639 bp *easI* encodes a protein with 212 amino acids. Figure S1 outlines the nucleotide sequence of the gene and its flanking sequences. The sequence, TAGTTT, at 27 nucleotides upstream of the start codon and the sequence, CTGTCC, located at 50 nucleotides upstream, are the putative −10 and −35 transcriptional elements, respectively. As suggested by Hawley and McClure (1983) on *E*. *coli* RNA polymerase σ^70^ consensus promoter analysis, the two consensus regions are separated by 17 nucleotides. A putative Shine‐Dalgarno site (AGGA) is located 8 bp upstream of the start codon. In addition, a putative *lux*‐box (TACTTTTTAAGTA) was found 81 bp upstream of the start codon. This palindromic sequence of *lux* box suggests that the putative LuxR homolog, EasR, may bind to the *easI* promoter to activate gene expression. However, this hypothesis requires further validation.

**Figure 3:**
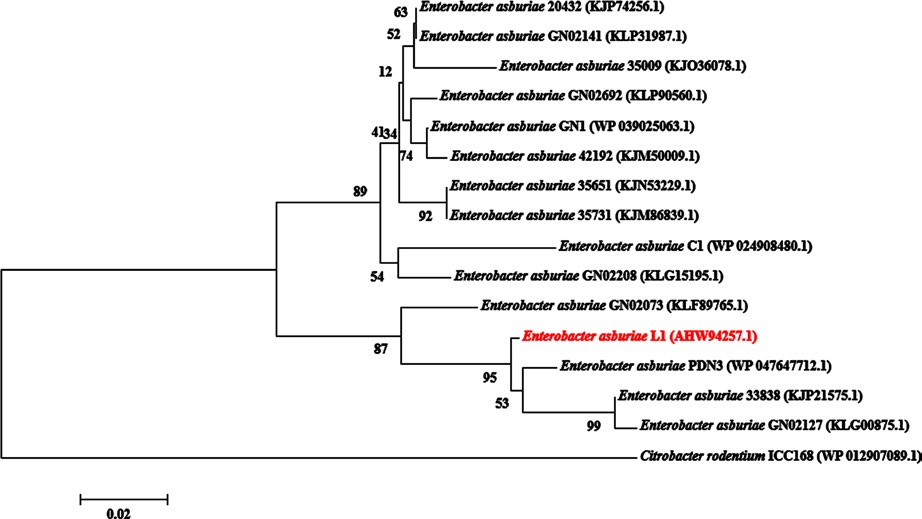
Phylogenetic tree showing the evolutionary distances between the putative AHL synthase, EasI of strain L1 (red word) with the other *E. asburiae* strains, generated using neighbor‐joining algorithm. The tree is drawn to scale, with branch lengths to show the evolutionary distances in the phylogenetic tree. The horizontal bar indicates evolutionary distance as 0.02 change per nucleotide position. The bootstrap values as percentage of 1,000 replications are given as numbers at the nodes. The LuxI homolog of *Citrobacter rodentium* is used as an outgroup

The phylogenetic analysis of LuxI homologs (Figure 3) shows the evolutionary distances between EasI and its counterparts from other *E*. *asburiae* strains, generated using neighbor‐joining algorithm. The multiple sequence alignment of the autoinducer protein sequences are illustrated in Figure S2. The alignment shows that EasI shares high similarities with other LuxI homologs of *E*. *asburiae* strains. It is worth noting that these LuxI family members contain the conserved 10 amino acid residues, characteristic of LuxI homologs (Parsek, Schaefer, & Greenberg, 1997).

### 3.3 Purification of EasI protein and SDS‐PAGE analysis

**Figure 4:**
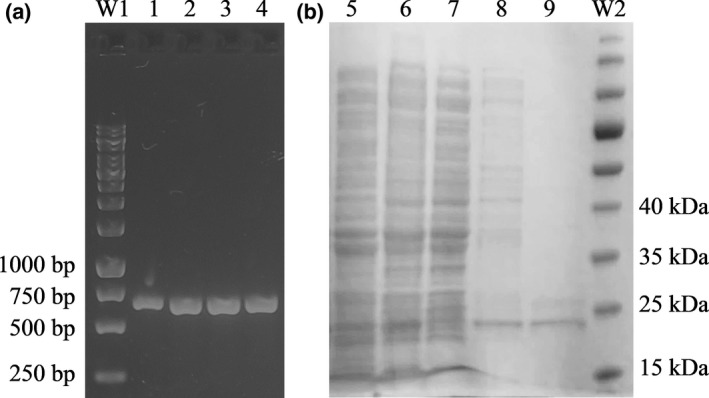
The amplified *easI* gene and the expressed His‐tagged protein. (a) Agarose gel electrophoresis of PCR results from genomic DNA of strain L1. Lanes 1 to 4 showed the 639 bp amplicons in replicates. (b) SDS‐PAGE electrophoretograms (under reducing conditions) of the purified recombinant EasI protein. Lane 5, cell lysates of noninduced *Escherichia coli* BL21 (DE3)pLysS harboring pET28a‐*easI*; Lane 6, cell lysates of induced *E*. *coli* BL21 (DE3)pLysS harboring pET28a‐*easI*, Lane 7, flow‐through fraction of purification step; Lane 8, wash fraction of purification step; Lane 9, eluted fraction containing recombinant EasI protein; Lane M1, 1 kb DNA ladder (Fermentas, Thermo Fisher Scientific, USA); Lane M2, protein marker (Fermentas, Thermo Scientific, USA) in kDa

The *easI* gene of strain L1 was amplified from the genomic DNA by PCR (Figure 4a). This 639 bp open reading frame (ORF) encodes an AHL synthase with a molecular mass (*M*_r_) of 24.9 kDa and isoelectric point (pI) of 6.07, as predicted from ExPASy server (Wilkins et al., 1999). The *easI* gene was cloned into pET28a overexpression vector, producing pET28a‐*easI*, with a 6× His‐tag residues. Expression of *easI* gene in *E*. *coli* BL21 (DE3)pLysS cells was overexpressed upon 1.0 mmol/L IPTG induction which has been tested to be the optimum concentration for induction (data not shown). The expressed His‐tagged EasI recombinant protein was later purified from cell lysate using Ni‐NTA metal‐affinity chromatography matrices. The purified protein has an estimated size which corresponds to the SDS‐PAGE profile (Figure 4b).

### 3.4 AHL detection and identification

**Figure 5:**
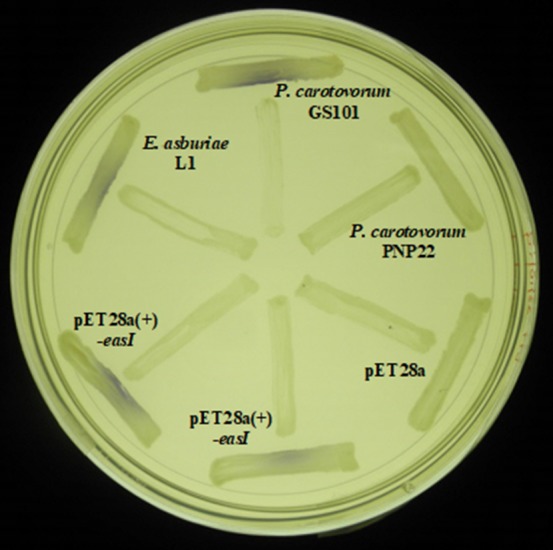
The detection of AHL using *Chromobacterium violaceum* CV026 cross streak. *Pectobacterium carotovorum* GS101 and *E. asburiae* L1 were used as positive controls, whereas *P. carotovorum* PNP22 and *E*. *coli* BL21 (DE3)pLysS harboring pET‐28a(+) were used as negative controls. The production of purple pigmentation by strain CV026 indicates the presence of short‐chain AHL molecule. The *easI* gene of L1 was successfully cloned into and expressed by *E*. *coli* BL21 (λDE3)pLysS

**Figure 6:**
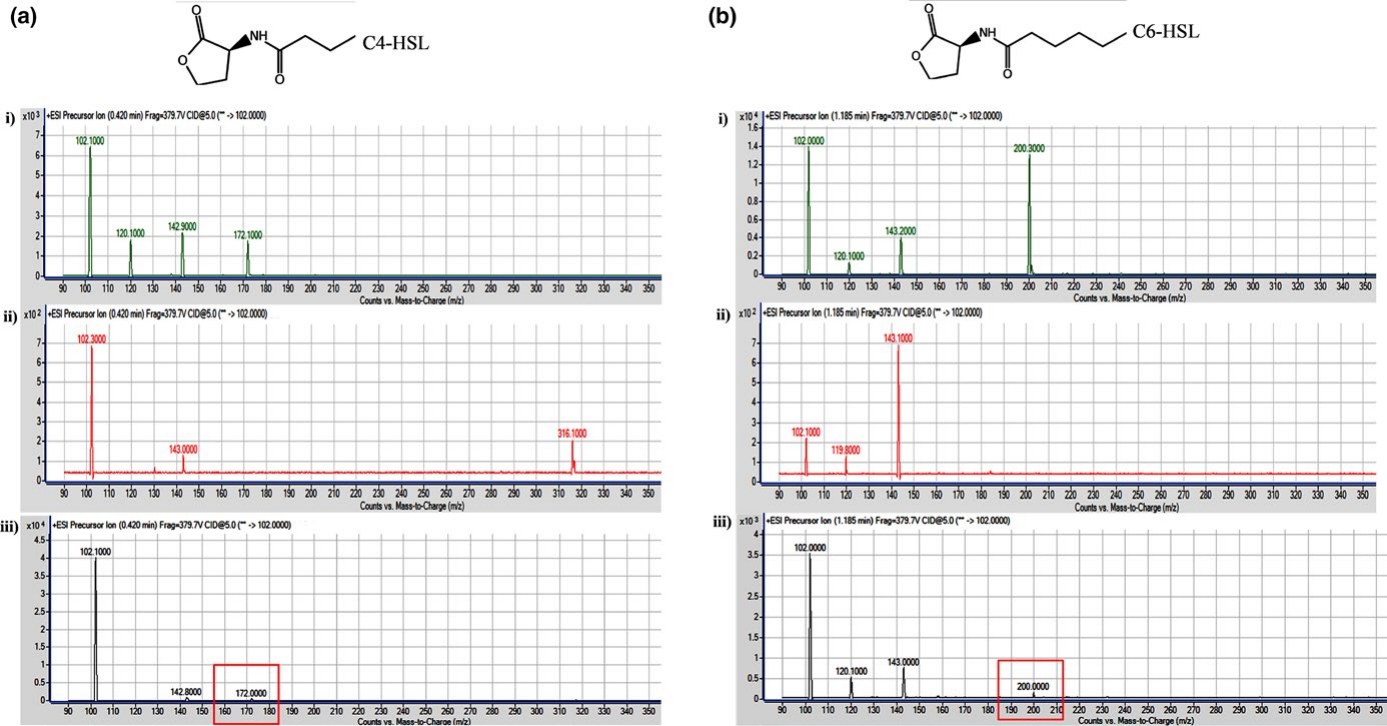
The MS analyses of the extracted AHLs from IPTG‐induced *E*. *coli* BL21 (DE3)pLysS harboring pET28a‐*easI*. The MS demonstrated the presence of (a) C4‐HSL (*m/z* 172.0000) and (b) C6‐HSL (*m*/*z* 200.0000) at the retention times 0.420 min and 1.185 min, respectively. (i) Mass spectra of synthetic AHL standards; (ii) Mass spectra of *E*. *coli* BL21 (DE3)pLysS harboring pET28a alone (control); (iii) mass spectra of induced *E*. *coli* BL21 (DE3)pLysS harboring pET28a‐*easI*

*Escherichia coli* BL21 (DE3)pLysS harboring pET28a‐*easI* was found to be AHL short‐chain producer as it activate the purple violacein production by *C*. *violaceum* CV026. As expected, the purple pigmentation was not observed for *E*. *coli* BL21 (DE3)pLysS harboring pET28a alone. Such characteristic was also displayed by the parental strain (Figure 5). LC–MS/MS system was then used to assess the AHL profile of the IPTG‐induced *E. coli* BL21 (DE3)pLysS harboring pET28a‐*easI*. The mass spectra (MS) analysis shows the presence of C4‐HSL (*m*/*z* 172.0000) and C6‐HSL (*m*/*z* 200.0000) (Figure 6) at retention time of 0.42 min and 1.185 min, respectively, from the bacterial spent culture supernatant. A fragment ion at *m*/*z* 102 was observed for each type of AHL, indicating the presence of the lactone ring of AHL. All the MS of the extracted AHLs were similar to their corresponding synthetic compounds at their respective retention times (Table S3). As for negative control, no AHL was found in the *E*. *coli* BL21 (DE3)pLysS harboring pET28a alone. These findings are in agreement with our previous study that showed the same AHL profile in the wild‐type strain L1 (Lau et al., 2013).

### 3.5 Biofilm formation and quantification in *E. asburiae* strain L1

**Figure 7:**
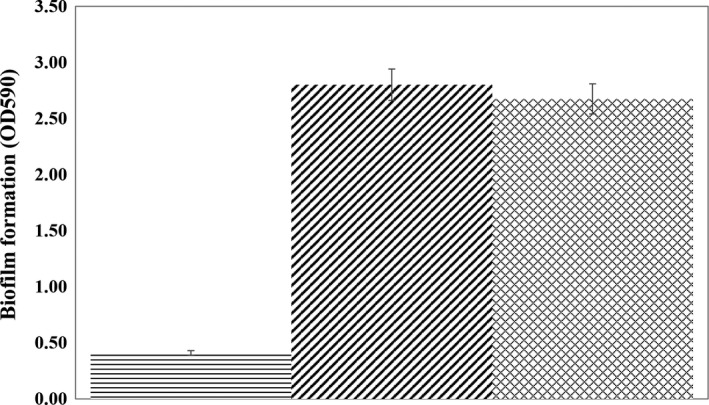
Qualitative analyses of biofilm formation in *E. asburiae* strain L1 (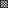). *P*. *aeruginosa* PAO1 (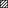) was used as the positive control, whereas blank LB medium (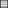) was used as negative control. Bars: 95% confidence intervals (IC_95_). The statistical significance was determined using unpaired *t* test (*p* < .001)

The biofilm‐forming characteristic in strain L1 was verified and quantified by a standard crystal violet binding assay. After 72 hr of growth at 37°C, quantification of biofilm in strain L1 was performed by comparison with a well‐known biofilm‐forming bacterium, *P*. *aeruginosa* PAO1. A detectable basal biofilm formation was found in strain L1, similar to strain PAO1 (Figure 7). This highly suggests the biofilm‐forming characteristic in strain L1.

## 4 DISCUSSION

Over the year, *Enterobacter* sp. have gained more attention as important and challenging pathogens (Sanders & Sanders, 1997), mainly due to their resistance toward a broad range of antimicrobial agents, like cefoxitin, a third‐generation cephalosporin, colistin, and aminoglycosides (Bouza & Cercenado, 2002; Paterson, 2006). Recently, a more concerned public health crisis is the emergence of Enterobacteria that produce *Klebsiella pneumoniae* carbapenemase (KPC‐type carbapenemases) (Kitchel et al., 2009; Nordmann, Naas, & Poirel, 2011; Tzouvelekis, Markogiannakis, Psichogiou, Tassios, & Daikos, 2012), which are highly resistant to different classes of antimicrobial agents and predominantly associated with nosocomial and systemic infections (Arnold et al., 2011). Although our understanding on the genus *Enterobacter* and its roles in human disease has increased throughout the years, the underlying complex mechanisms of pathogenicity of different *Enterobacter* sp. is yet to be discovered. There is very little information concerning the AHL‐type QS mechanism in *Enterobacter* sp. In fact, whether the virulence factors in *Enterobacter* spp. is controlled by this system remains an unsolved mystery. Nonetheless, AHL‐type QS activities are proven to control virulence factors in other species such as *Burkholderia cepacia*,* Agrobacterium tumefaciens,* and *Erwinia carotovora* (de Kievit & Iglewski, 2000). Therefore, in the early stage, we postulated that our current findings of QS activity in strain L1 (Lau et al., 2013) are related to the regulation of virulence factors. This brought us the interest to further investigate and expand our studies toward QS properties of *E*. *asburiae*.

In this study, the complete genome of strain L1 was further analyzed and comparative genomic analysis was conducted with its closest sequenced relatives. Among these strains, GN02073, GN02127, and 33838 are KPC‐producing strains that were isolated from human source, whereas PDN3 was available from *Populus* root. Although strains L1 and PDN3 were isolated from phyllosphere environment, their genomes share high similarities with *E*. *asburiae* clinical strains, with more than 95%. This strongly indicates a close relationship among these bacterial strains and suggested strain L1 may possess some virulence factors that are similar to the clinical strains. In addition, RAST analysis revealed the absence of heme, hemin uptake, and utilization subsystems in strains L1 and PDN3. This possibly suggests that strains L1 and PDN3 could possibly depend on the high‐affinity iron‐binding molecules (i.e., siderophore) to scavenge iron from its environment. This iron gathering ability of siderophore is especially important for bacteria as etiologic agent in disease and infection. A study by Wen, Kim, Son, Lee, and Kim (2012) reported that siderophore production is regulated by cell density. Numerous studies have shown that QS regulation of siderophores is associated with the production of virulence factors and biofilm‐forming activity of *P*. *aeruginosa* (Lamont, Beare, Ochsner, Vasil, & Vasil, 2002; Patriquin et al., 2008; Rutherford & Bassler, 2012). Besides, the study by Modarresi et al. (2015) showed that during iron limitation stage, the activities of AHL and siderophore production of *Acinetobacter baumannii* were enhanced. This allows the bacterial cells to survive under inappropriate environment conditions (e.g., low concentration of micronutrients) and form strong biofilm. To date, correlation between QS and siderophore in *Enterobacter* spp. has never been explored. However, production of AHL and siderophore in other bacterial groups have revealed their correlation with biofilm formation. Therefore, our current finding of siderophore in this QS strains might be a stepping stone to unravel the exact roles of QS on siderophore production, especially in *E. asburiae*.

We further elucidated the genome of strain L1 by focusing on the CDS that play a role in cell‐to‐cell communication system in *Enterobacter* sp. to shed some light on the roles of QS system. In silico analysis of the *luxI* gene cluster among strain L1 and its closely related strains showed a highly conserved *luxI/R* gene clusters. The GCN5‐related N‐acetyltransferase and acetyltransferase GNAT families, which are located in the vicinity of the *luxI/R* genes, play important roles in fatty acid synthesis (Williams, Winzer, Chan, & Cámara, 2007; Xie, Zeng, Luo, Pan, & Xie, 2014). Apart from that, a signal‐recognition protein, sensor histidine kinase, is found downstream of every *luxI* homolog. Studies have shown that this protein is essential in many aspects of bacterial physiology, including bacterial infections, by facilitating the bacteria to sense environmental or cellular stimuli and alter its cytoplasmic autokinase activity in response to these signals (Bader et al., 2005). In fact, a study by Ferrieres and Clarke (2003) reported that RcsC sensor histidine kinase is needed for normal biofilm development in *E*. *coli*.

Biofilms are vital environmental reservoirs for pathogens. In fact, this growth mode may grant organisms with survival benefits in natural environments and increase their virulence (Parsek & Singh, 2003). Additionally, the occurrence of the pathogenic traits during chronic infection is often connected to the ability of microorganisms to produce biofilms (Bjarnsholt, 2013; Wolcott & Ehrlich, 2008). Besides, biofilm formation on food surfaces and food‐processing equipment is a persistent downside within the food industry, leading to serious health issues and inflicting great economic loss (Bai & Rai, 2011; Kumar & Anand, 1998). This phenotype is a cooperative group behavior that involves bacterial populations living embedded during a self‐generated extracellular matrix, which provides resistance toward antimicrobial substances (Costerton, Lewandowski, Caldwell, & Lappin‐Scott, 1995; Skandamis & Nychas, 2012). By this, the bacteria which possess biofilm will have higher food spoilage and virulence potential.

Our current work using crystal violet binding assay revealed the biofilm‐forming characteristic in strain L1. To date, no literature has revealed the association of QS and biofilm formation in *E*. *asburiae* as well as other *Enterobacter* spp. However, biofilm development and QS have been reported as closely interconnected processes in other genera (Solano, Echeverz, & Lasa, 2014). For example, it has been reported that one of the members of Enterobacteriaceae, *Hafnia alvei* 071, which is also a common bacterial food contaminant, synthesizes AHL molecules under the direction of the *luxI* homolog, *halI*. The AHLs were found to be important in biofilm formation as such phenotype was found to be impaired in *H. alvei* 071 *halI* mutant (Bai & Rai, 2011; Viana, Campos, Ponce, Mantovani, & Vanetti, 2009). Apart from that, a research by Davies et al. (Davies et al., 1998) also discovered that cell‐to‐cell signaling molecules are involved in the biofilm development of *P. aeruginosa*. It was found that the *lasI* mutant of *P*. *aeruginosa* resumed its normal biofilm activity in the presence of synthetic signaling molecules. Based on previous studies, the biofilm‐forming characteristic in strain L1 is highly postulated to be a QS‐dependent phenotype in strain L1. As such, there is a need to pay more attention on the relationship between QS and biofilm formation characteristic in *E*. *asburiae*, in order to shed some light on the mechanisms of infections caused by *E*. *asburiae*.

The study on QS has been of great interest as it is one of a suitable approach to understand the regulated phenotype such as biofilm formation and virulence (Rutherford & Bassler, 2012; Singh, 2015). Over the years, a large number of LuxI homologs have been found (Case, Labbate, & Kjelleberg, 2008). However, by knowing the sequence alone will not help to elucidate the type of AHLs produced by a given LuxI and predict the nature of AHLs produced. In this study, the *easI* gene from *E*. *asburiae* strain L1 has been cloned and characterized. Heterologously expressed EasI protein activated AHL biosensor *C*. *violaceum* CV026, indicating this EasI is a functional AHL synthase. The production of C4‐HSL and C6‐HSL from spent culture supernatant of induced *E*. *coli* BL21 (DE3)pLysS harboring the recombinant EasI was confirmed by LC–MS/MS analysis, suggesting that EasI is undoubtedly the AHL synthase of strain L1. Interestingly, C4‐HSL was found present in a higher amount than C6‐HSL by *E*. *coli* harboring *easI* from the mass spectra analysis (Figure S3).

Since the function of AHL synthase in *E*. *asburiae* has never been explored, we have compared the EasI protein homolog to other bacteria with similar QS protein. Our analysis showed that the EasI protein has highest similarity to the AHL synthase, CroI, from *Citrobacter rodentium* (77% identity), followed by PagI from *Pantoea agglomerans* (73% identity), SmaI from *Serratia* sp. ATCC 39006 and SwrI from *Serratia liquefaciens* MG1 (46% identity). Several studies have shown that these LuxI homologs also produce C4‐HSL as the major AHL and C6‐HSL as the minor AHL (Barnard et al., 2007; Coulthurst et al., 2007). In *C*. *rodentium*, the *croI* mutant was reported to possess a weaker adhesive ability to an abiotic surface than the wild‐type strain (Coulthurst et al., 2007). Besides, the production of C4‐HSL by *S*. *liquefaciens* MG1 was found to play an important role for normal biofilm development. Their study mentioned that mutants of *S*. *liquefaciens* MG1 that was incapable of producing AHL formed a thin and nonmature biofilm lacking cell aggregates (Labbate et al., 2004). Apart from that, Barnard et al. (2007) have proven that SmaI was found to regulate the carbapenem production, prodigiosin, and virulence factor production in strain ATCC 39006. On the other hand, gall‐forming plant pathogen, *P*. *agglomerans* produced C4‐HSL as a major and C6‐HSL as a minor QS signal to regulate its gall formation on plants. The loss of QS regulation in this strain led to reduction in the gall size (Chalupowicz et al., 2008). In another Enterobacteriaceae *Serratia* sp. strain 12, the production of C4‐HSL and C6‐HSL is needed for hemolytic activity, swarming motility, and synthesis of chitinase (Coulthurst, Williamson, Harris, Spring, & Salmond, 2006). In other gammaproteobacterium, *Aeromonas hydrophila* was also found to synthesize C4‐HSL in large amount compared to C6‐HSL. The LuxI and LuxR homologs play essential role in biofilm formation and virulence determinant such as exoprotease production and Type IV secretion system (Khajanchi et al., 2009). Therefore, although the regulatory roles of EasI from strain L1 could be predicted based on previous findings, further investigation is required to truly understand how the QS mechanism works in mediating the virulence factors production in this bacterium.

Although LuxI/R QS systems have been widely studied in various bacteria, there is still limited knowledge on QS activity in *Enterobacter* sp. and most studies place great emphasis on clinical isolates. Hence, our study on the QS system in *E*. *asburiae* strain L1 has provided a great stepping stone to unravel the roles of the bacterium in different environments. Besides, this first documentation of the cloning and characterization of *easI* gene facilitates the elucidation of the exact role and regulatory molecular mechanism of QS system in this bacterium. This work enables mutant construction with defective *easI* for genome‐wide comparative transcriptomics study to further investigate the roles of AHLs produced by strain L1. In future, it is also an interest to explore interaction between LuxI homolog and compounds exhibiting anti‐QS properties as one of the way to control virulence of the bacterium.

## CONFLICTS OF INTEREST

The authors declare no conflict of interest.

## DNA DEPOSITION

The following information was supplied regarding the deposition of DNA sequences: Genbank, accession no. CP007546, AHW94257.1, and AHW94256.1.
