# Supplementary material for: Cloning and characterization of short‐chain N‐acyl homoserine lactone‐producing Enterobacter asburiae strain L1 from lettuce leaves
Source: Microbiologyopen. 2018 Jul 7;7(6):e00610. doi: 10.1002/mbo3.610 (PMC6291789; doi:10.1002/mbo3.610)
Supplement: Supplementary file 1 [file MBO3-7-e00610-s001.docx]

**Supplementary Materials**

**Table S1.** Bacterial strains and plasmids used in this study

| Strain | | Genotype / Description | Source / Reference |
| --- | --- | --- | --- |
| *Enterobacter asburiae* L1 | | Lettuce isolate. QS strain with the formation of purple violacein pigment in the presence of short chain exogenous AHL molecules. | This study |
| *Chromobacterium violaceum* CV026 | | A biosensor with mini-Tn5 mutant derived from *C. violaceum* ATCC 31532. In the presence of short chain exogenous AHL molecules, it triggers the purple violacein pigment. | McClean et al., 1997 |
| *Pectobacterium carotovorum* GS101 (previously known as *Erwinia carotovora*) | | Acts as positive control for biosensors. It produces short chain AHLs to activate biosensor *C. violaceum* CV026. | Jones et al., 1993 |
| *Pectobacterium carotovorum* PNP22 | | Acts as negative control for biosensors. It does not produce AHL molecules to activate biosensor *C. violaceum* CV026. | Jones et al., 1993 |
| *Escherichia coli* DH5α | | dl*ac*Z∆M15 ∆(*lacZYA-argF*)U169 *rec*A1 *end*A1 hsdR17(*rK^-^mK^+^*) *supE*44 *thi-1 gyrA96 relA1*. | Invitrogen, USA |
| *Escherichia coli* BL21 (DE3)pLysS | | F^-^ *ompT gal dcm lon hsdS_B_(r_B_^-^ m_B_^-^)* λ(DE3 [*lacI* *lacUV5*-*T7p07* *ind1* *sam7* *nin5*]) [*malB*^+^]_K-12_(λ^S^) pLysS[*T7p20* *ori*_p15A_]; Cm^R^. | Novagen, Germany |
| *Pseudomonas aeruginosa*  PAO1 | | Ubiquitous environmental bacterium that is an opportunistic nosocomial pathogen. A well-known biofilm forming bacterium. | Chan et al., 2011 |
| Plasmids | | **Genotype / Description** | **Source / Reference** |
| pGEM^®^-T | Toxin-antitoxin (TA) cloning vector, Amp^R^. | | Promega, USA |
| pGEM^®^-T -*easI* | pGEM^®^-T containing 639 bp of *easI* with NcoI-BamHI sites; Amp^R^. | | This study |
| pET-28a(+) | Circle plasmid carrying N-terminal His-tag / thrombin / enterokinase configuration plus an optional C-terminal His-tag sequence; Km^R^. | | Novagen, Germany |
| pET-28a(+)-*easI* | pET-28a(+) containing 639 bp of *easI* cloned into NcoI-BamHI sites; Km^R^. | | This study |

**Table S2.** Genomic coverage of several major subsystems found in *E. asburiae* strain L1 and its closest relatives [*E. asburiae* PDN3 (JUGH00000000.1), *E. asburiae* GN02073 (LDCE01000001.1), *E. asburiae* 33838 (LAAP00000000.1) and *E. asburiae* GN02127 (LDCH00000000.1)]. The number of subsystem feature counts of each strain is shown in number.

| Subsystems | *Enterobacter asburiae* L1 genes | *Enterobacter asburiae* PDN3 genes | *Enterobacter asburiae* GN02073 genes | *Enterobacter asburiae* 33838 genes | *Enterobacter asburiae* GN02127 genes |
| --- | --- | --- | --- | --- | --- |
| Amino acids and derivatives | 471 | 487 | 462 | 478 | 483 |
| Carbohydrates | 593 | 582 | 565 | 606 | 589 |
| Cofactors, vitamins, prosthetic groups and pigments | 252 | 267 | 267 | 272 | 269 |
| Protein metabolism | 246 | 293 | 263 | 263 | 267 |
| RNA metabolism | 189 | 213 | 211 | 234 | 234 |
| Fatty acids, lipids and isoprenoids | 121 | 134 | 134 | 138 | 135 |
| Membrane transport | 199 | 201 | 197 | 206 | 227 |
| Cell wall and capsule | 229 | 217 | 212 | 226 | 220 |

**Table S3.** The molecular mass (*m/z*) and retention time showed by the synthetic AHLs and identified AHLs from cell culture supernatant.

| **Analyte** | **Mass (*m/z*)** | **Retention time (min)** |
| --- | --- | --- |
| Synthetic standard C4-HSL | 172.1000 | 0.420 |
| Sample containing C4-HSL | 172.0000 | 0.420 |
| Synthetic standard C6-HSL | 200.3000 | 1.185 |
| Sample containing C6-HSL | 200.0000 | 1.185 |


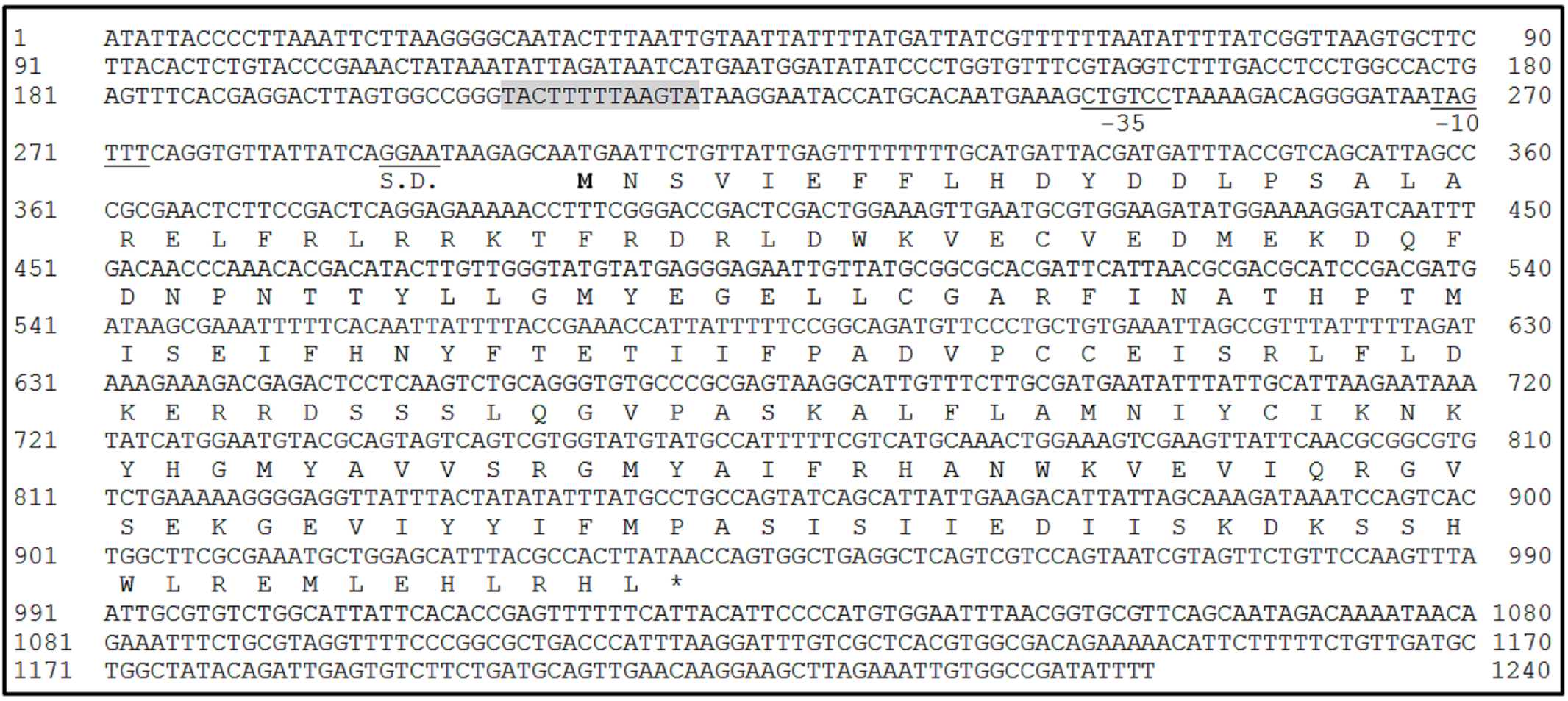


**Figure S1.** The nucleotide sequence of *easI* gene of *E. asburiae* strain L1 and its flanking sequences. Single letters which code for deduced amino acid sequence are shown below the nucleotide sequence. The stop codon (TAA) is marked by an asterisk. The translational start site (M) is in bold and S.D. denotes the putative Shine-Dalgarno site. The proposed core promoter elements, -10 and -35 boxes, are underlined. A putative *lux* box is highlighted in grey.

**
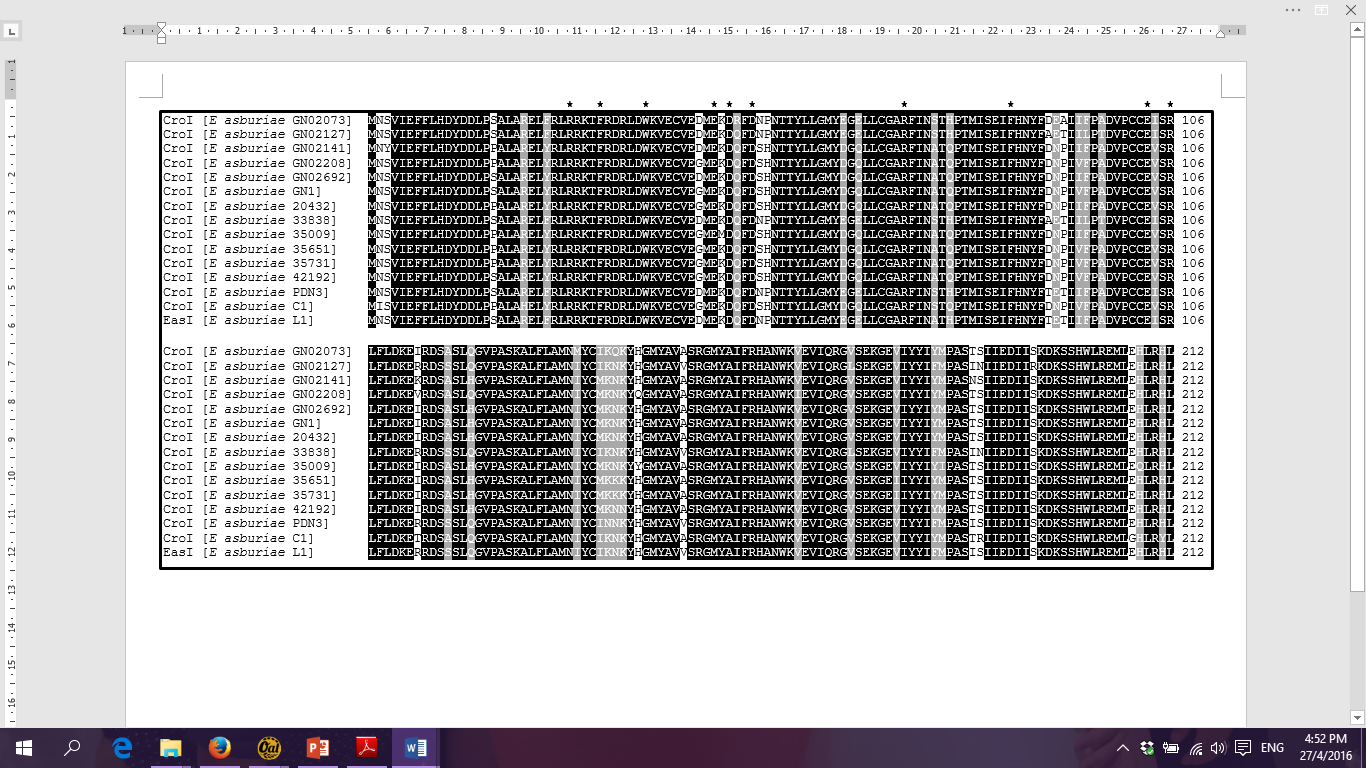
**

**Figure S2.** CLUSTAL O (1.2.0) multiple sequence alignment of *N*-acyl homoserine-lactone (AHL) autoinducer protein sequences of *E. asburiae* strain L1 with protein sequences from other *E. asburiae* strains. Sequences were derived from NCBI database (http://www.ncbi.nlm. nih.gov) and were aligned using CLUSTAL OMEGA software (http://www.ebi.ac.uk). The 10 invariant amino acids characteristics of LuxI homologues are denoted with asterisks. Residues that are identical among the sequences are given a black background while those that are highly similar among the sequences are given a grey background. GenBank accession numbers in parentheses: CroI *E. asburiae* GN02073 (KLF89765.1), CroI *E. asburiae* GN02127 (KLG00875.1), CroI *E. asburiae* GN02141 (KLP31987.1), CroI *E. asburiae* GN02208 (KLG15195.1), CroI *E. asburiae* GN02692 (KLP90560.1), CroI *E. asburiae* GN1 (WP_039025063.1), CroI *E. asburiae* 20432 (KJP74256.1), CroI *E. asburiae* 33838(KJP21575.1), CroI *E. asburiae* 35009 (KJO36078.1), CroI *E. asburiae* 35651 (KJN53229.1), CroI *E. asburiae* 35731 (KJM86839.1), CroI *E. asburiae* 42192 (KJM50009.1), CroI *E. asburiae* PDN3 (WP_047647712.1), CroI *E. asburiae* C1 (WP_024908480.1).


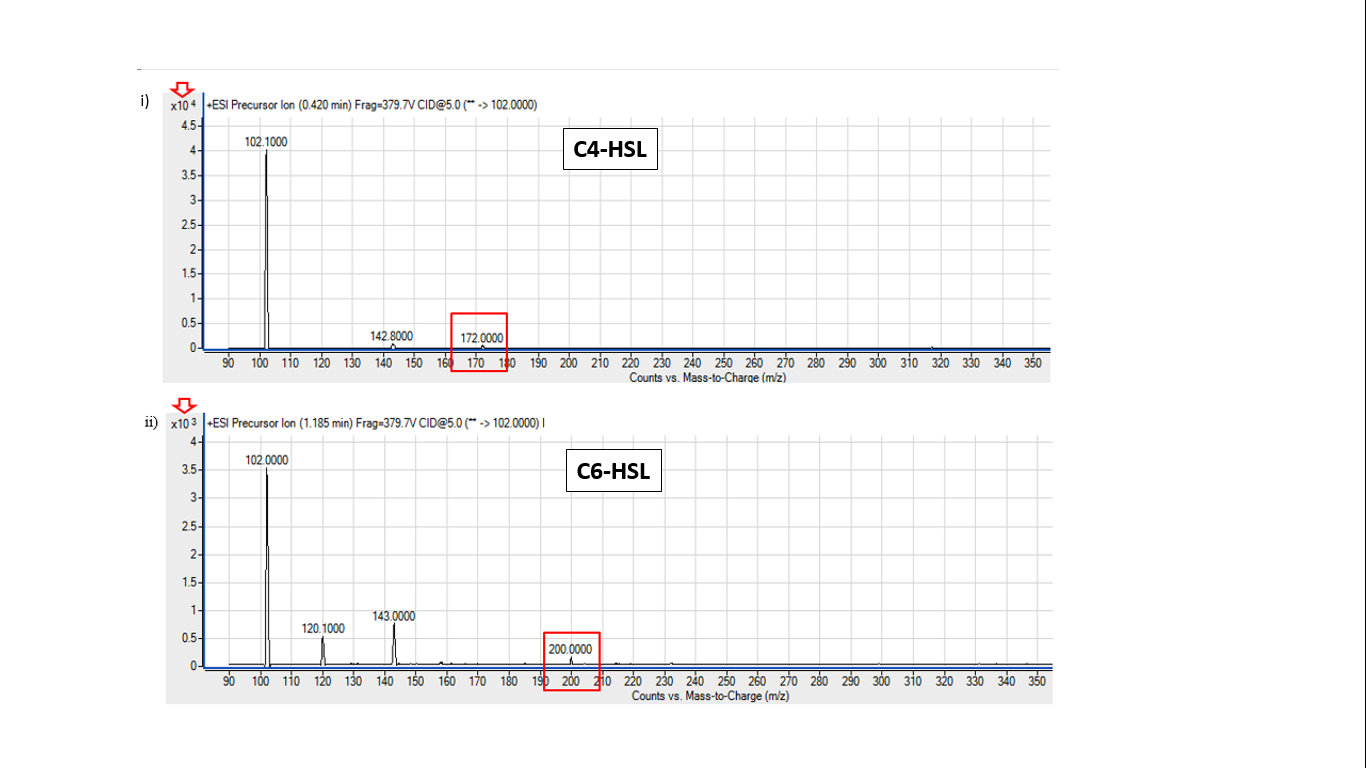


**Figure S3.** Enlarged mass spectra of cell supernatant of *E. coli* BL21 harboring pET28a-*easI* showing the presence

of C4-HSL and C6-HSL at their respective retention time.
